# Racial and ethnic disparities post-hospitalization for COVID-19: barriers to access to care for survivors of COVID-19 acute respiratory distress syndrome

**DOI:** 10.1038/s41598-024-61097-0

**Published:** 2024-05-21

**Authors:** Alicia Cañas, Allison Wolf, Angela Mak, Jacob Ruddy, Sal El-Sadek, Laura Gomez, David Furfaro, Robert Fullilove, Kristin M. Burkart, Jennifer Zelnick, Max R. O’Donnell

**Affiliations:** 1 Department of Medicine, Columbia University Medical Center, New York City, USA; 2 Division of Pulmonary, Allergy, and Critical Care Medicine, Columbia University Medical Center, New York City, USA; 3 School of Global Health, Dahdaleh Institute of Global Health Research, York University, Toronto, Canada; 4 Department of Epidemiology, Mailman School of Public Health, Columbia University Medical Center, New York City, USA; 5 Division of Pulmonary, Allergy, and Critical Care Medicine, Beth Israel Deaconess Medical Center, Brookline, MA USA; 6 Department of Sociomedical Sciences, Mailman School of Public Health, Columbia University Medical Center, New York City, USA; 7 Graduate School of Social Work, Touro University, New York City, USA; 8 Division of Pulmonary, Allergy, and Critical Care Medicine, Department of Epidemiology, Columbia University Medical Center, Suite E101, 8th Floor, PH building, 622 W. 168th street, New York City, NY 10032 USA

**Keywords:** COVID-19, Long COVID, ARDS, Racial and ethnic disparities, Access to care, Risk factors, Viral infection

## Abstract

Racial and ethnic health disparities in the incidence and severity of Coronavirus Disease 2019 (COVID-19) have been observed globally and in the United States. Research has focused on transmission, hospitalization, and mortality among racial and ethnic minorities, but Long COVID-19 health disparities research is limited. This study retrospectively evaluated 195 adults who survived COVID-19 associated acute respiratory distress syndrome (C-ARDS) in New York City from March–April 2020. Among survivors, 54% met the criteria for Long COVID syndrome. Hispanic/Latinx patients, were more likely to be uninsured (p = 0.027) and were less frequently discharged to rehabilitation facilities (p < 0.001). A cross-sectional telephone survey and interview were conducted with a subset of survivors (n = 69). Among these, 11% reported a lack of follow-up primary care post-discharge and 38% had subsequent emergency room visits. Notably, 38% reported poor treatment within the health care system, with 67% attributing this to racial or ethnic bias. Thematic analysis of interviews identified four perceived challenges: decline in functional status, discrimination during hospitalization, healthcare system inequities, and non-healthcare-related structural barriers. Sources of resilience included survivorship, faith, and family support. This study highlights structural and healthcare-related barriers rooted in perceived racism and poverty as factors impacting post-COVID-19 care.

## Introduction

As of September 2023, more than 6.3 million persons with Coronavirus Disease 2019 (COVID-19) have been hospitalized in the United States with over 1.14 million deaths^1^. Data from geographic locations reporting cases by race and ethnicity shows that historically vulnerable communities have experienced a disproportionate burden of disease. Non-Hispanic Blacks and Hispanic/Latinos are 1.7 and 1.8 times more likely to die from COVID-19 than whites; and 2.4 times more likely to be hospitalized^2^. Structural determinants of health including racism and discrimination, access to healthcare and health resource utilization, occupation, education, income, and place of residence are all factors associated with higher incidence and severity of COVID-19 disease among minority groups^3^. As an illustrative example, a large systematic review and meta-analysis found that racial disparities in COVID-19 incidence and intensive care unit (ICU) hospitalization substantially decreased when adjusting for area deprivation index and clinical care quality^4^. While there is a substantial body of literature describing the association of race and ethnicity with risk for development of severe COVID-19 and COVID-19 associated mortality^5–8^, fewer studies have explored post-hospitalization disparities in functional outcomes and access to follow-up care^9^, particularly in patients with persistent COVID-19 associated symptoms, termed Long COVID.

The main indication for ICU admission related to severe COVID-19 is need for mechanical ventilation secondary to the acute respiratory distress syndrome (ARDS)^10–13^. ARDS is defined as a syndrome of severe hypoxemia despite positive pressure respiratory support, bilateral infiltrates on chest radiograph, and exclusion of heart failure as the primary cause for these clinical signs. Despite temporal trends towards improved outcomes for critically ill patients, and the development of effective immunomodulatory treatments, mortality for patients with COVID ARDS (C-ARDS) remains high^14–16^. Mortality rates vary unpredictably with viral surges, and are highest in resource-limited settings^17,18^. Although prognostic factors in severe COVID-19 have been identified (age, obesity, co-morbidities including diabetes and renal dysfunction), the biological determinants of prognosis and treatment response are incompletely understood^17,19,20^.

Long COVID, also known as ‘post-acute sequelae of COVID-19’, is a condition affecting multiple organ systems, characterized by persistent, often intense symptoms after a SARS-CoV-2 infection. Among ambulatory adults with COVID-19, the estimated incidence of Long COVID varies between 7.5 and 41%. Although expert opinion suggests increasing risk for Long COVID with increased disease severity, and C-ARDS is the most severe presentation of COVID-19 pneumonia, there have been few articles on the risks for Long COVID post C-ARDS^21,22^.

Prior critical care literature has described longstanding physical, cognitive, and neuropsychiatric sequelae among ICU survivors, collectively referred to as the “post-intensive care syndrome” (PICS)^23–25^. Prolonged hospitalization in the ICU often leads to extensive rehabilitation needs and discharge to specialized rehabilitation units rather than to home. Evidence shows racial/ethnic variations in long-term acute care hospital and rehabilitation unit use after critical illness^26,27^, with non-Hispanic Blacks and Hispanic/Latino patients being more likely to be discharged to home rather than to a facility^27^. When adjusting for insurance status, this difference largely disappears, suggesting insurance is a major driver of post-acute care rehabilitation use^25,27^. Given the close relationship between insurance status, race/ethnicity, and hospital disposition, we expect worse health outcomes and access challenges among traditionally vulnerable groups in the post-acute care setting.

In New York City, where high proportions of COVID-19 cases early in the pandemic required ICU admission and mechanical ventilation^28^, we anticipate long-term disability will be substantial and may disproportionally affect racial and ethnic minorities. We hypothesized that post-COVID-19 disability is compounded by systemic racism and structural barriers to care for C-ARDS survivors in New York City.

## Methods

### Study design and population

To examine this hypothesis, we utilized a mixed methods approach with two components: (1) an electronic medical record (EMR)-based retrospective cohort study nested in a previously published study of C-ARDS and (2) a cross-sectional telephonic survey of prospectively identified C-ARDS survivors with quantitative and qualitative components.

Using a cohort defined in a prior study of C-ARDS^29^, we identified adult patients hospitalized at Columbia University Irving Medical Center from March 1st through April 30th 2020, with a positive RT-PCR for severe acute respiratory syndrome coronavirus 2 (SARS-COV-2), who developed severe hypoxemic respiratory failure requiring mechanical ventilation meeting Berlin criteria for ARDS and who were subsequently discharged from the hospital without need for ventilator support^30^. Patients who received a tracheostomy and were weaned off the ventilator prior to discharge were included. Exclusion criteria included transfer from another institution, tracheostomy prior to admission, in-hospital death, and discharge with a ventilator.

Using EMR, we collected demographic and clinical characteristics including data on follow-up appointments, emergency room visits, and re-hospitalization within one year of discharge. Long COVID was defined using CDC and WHO definitions at 3–8 months post-index COVID-19 hospital discharge. Prior conditions were considered to prevent misclassification. Long COVID defining symptoms, date of clinical definition and type of clinical encounter were recorded.

All C-ARDS survivors were contacted using a mailing to allow for opt-out from study participation. Subsequently, three researchers in the group (A.C., L.G., S.E.) attempted to contact all potential survey participants via telephone. Up to three attempts were made to contact each participant between July and November 2021. We obtained verbal informed consent using a standardized script prior to administering the survey. In the case of Spanish-speaking participants, a native Spanish speaker administered the survey in Spanish. Given the nature of our patient population, we expected significant disability within our cohort and allowed adult caregivers to answer questions on behalf of the participant as deemed appropriate. We confirm that all research was performed in accordance with relevant guidelines/regulations. The Institutional Review Board of Columbia University Irving Medical Center approved this study.

### Questionnaire construction

Researchers in the group developed a survey derived from the Everyday Discrimination Scale adapted for the health care setting^31,32^, including 33 close-ended questions and 4 open-ended questions addressing functional status, socioeconomic status, perceived discrimination, and access to healthcare after COVID-19 hospitalization (Supplement 1). The survey was translated into Spanish by a certified translator.

### Statistical methods

Descriptive statistics were used to describe demographic data and analyze survey results. Differences among subgroups were evaluated with student *t* test, ANOVA, and chi-square tests. Survival analysis was calculated from time of intubation. Tests between strata were assessed using log-rank test. Bivariate analysis was used to assess the relationship between exposure variables and outcomes. Variables with ≥ 10% change in effect size and/or p value < 0.05 were included in the multivariable model. Statistical analysis was performed by using SAS version 9.4 software (SAS Institute, Cary, NC, USA).

### Qualitative methods

Post-discharge challenges were contextualized via analysis of telephonically collected interview data derived from open-ended questions. Telephonic interviews were transcribed, anonymized, translated, and subjected to thematic analysis by the study investigators (J.Z., E.C.C., A.C.). Three researchers separately reviewed qualitative data, extracted themes, and reconciled differences. A coding scheme categorized themes under broader concepts through discursive techniques to discern patterns, evaluate context, and attain conceptual clarity. Emerging themes underwent interrogation via reflexive practice and systematic consultations to reinforce analytic dependability, confirmability, and trustworthiness^33^. Quotes were then recoded with participant numeric identifier, race, ethnicity, and gender to allow for further analysis of commonalities. Qualitative analysis was guided using the concept of racism as a social determinant of health^34^, modified by COVID-19 specific considerations.

## Results

**Figure 1:**
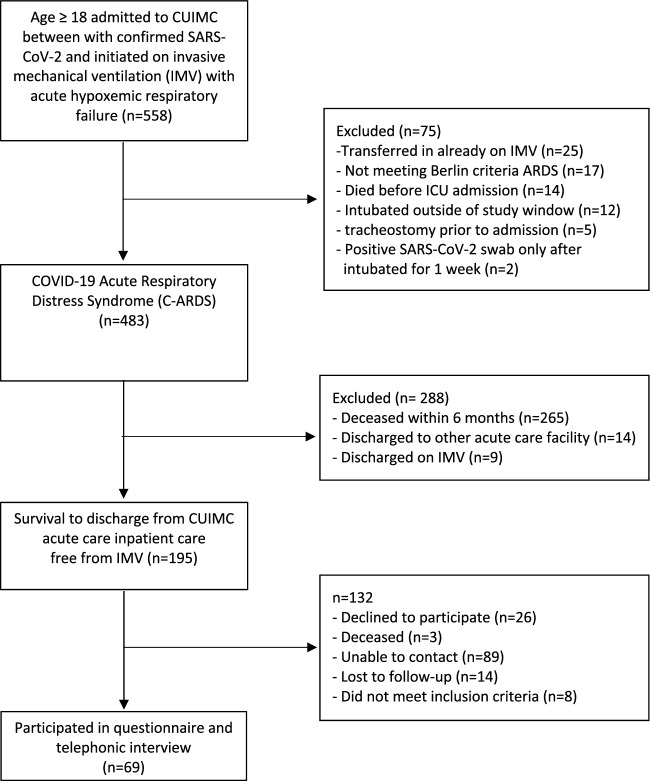
Study flow diagram.

483 patients with C-ARDS met initial study criteria and were included in survival analysis. Median PaO2:FiO_2_ ratio on the day of intubation was 138 (IQR 91–197) and median compliance was 23.5 (IQR 18.8–30.6). 195/477 (41%) survived after a median hospitalization of 41.5 days (IQR 25–59) and were discharged from hospital not requiring mechanical ventilation (Fig. 1). Survivors included 115 (58%) Hispanic/Latinx, 34 (18%) non-Hispanic Black, and 17 (9%) non-Hispanic White patients (Table 1). In an adjusted regression model, age, male gender, and C-reactive protein, but not race/ethnicity significantly predicted survival free of invasive mechanical ventilation (IMV) (Table S1). Hispanic/Latinx patients were more likely to be uninsured (p = 0.027) and discharged to home rather than to rehabilitation (p < 0.001).

**Table 1:** Baseline characteristics COVID-19 acute respiratory distress syndrome (C-ARDS) cohort stratified by invasive mechanical ventilator (IMV)-free survival.

Variables		Total cohort (N = 483)	Survivors to IMV-free discharge (N = 195)
Age, median (IQR)		65 (55–74)	59 (47.5–66.5)
Gender	Female	164 (34%)	64 (33%)
	Male	319 (66%)	131 (67%)
Race/Ethnicity	Black*	86 (18%)	34 (18%)
	White*	56 (12%)	17 (9%)
	Hispanic/Latinx	285 (59%)	115 (58%)
	Other	56 (11%)	29 (15%)
Body mass index, median (IQR)		30.2 (25.5–33.1)	28.9 (26.1–33)
Smoker (current or former)		98 (21%)	35 (18%)
Diabetes		210 (44%)	67 (34%)
Hypertension		328 (69%)	106 (54%)
Coronary arterial disease		119 (25%)	21 (11%)
Symptom duration, days (IQR)		7 (4–9)	7 (4–9)
Median length of stay, days (IQR)		26 (10–48)	45 (45–63)
PaO_2_/FiO_2_ ratio, median (IQR)		138 (91–197)	153 (97–200)
C-reactive protein, median (IQR)		232 (143–300)	257 (162.4–300)
Diabetes		210 (44%)	67 (34%)

*Non-Hispanic

Patients were evaluated for Long COVID at 3–6 months post-C-ARDS admission, using standard definitions as described above through EMR. Of the 128 patients evaluable, 53.1% met criteria for Long COVID (Table 2). Defining symptoms were predominantly dyspnea (43%), deconditioning (40%), chronic cough (24%), and fatigue (13%). In our cohort, there was no difference in proportion diagnosed with Long COVID by race or ethnicity.

**Table 2:** C-ARDS survivors meeting criteria for long Covid.

	Meeting criteria for long COVID (n = 68)	Not meeting criteria for long COVID (n = 60)
Age, median (IQR)	59 (51–67)	62 (46–67)
Gender (%)		
Female	16 (24)	21 (35)
Male	52 (76)	39 (65)
Race/ethnicity (%)*		
Black	19 (33)	15 (33)
White	15 (26)	8 (17)
Other	23 (40)	23 (50)
Hispanic/Latinx	38 (66)	36 (75)
Body mass index, median (IQR)	29 (26–36)	29 (26–33)
Diabetes (%)	17 (25)	27 (45)
Hypertension (%)	30 (44)	31 (52)
Coronary artery disease (%)	9 (13)	7 (12)
Symptom duration prior to admission, days (IQR)	7 (5–10)	7 (4–8)
Median length of stay, days (IQR)	51 (33–69)	47 (33–61)
PaO2/FiO2 Ratio, median (IQR)	133 (84–185)	152 (112–199)
C-reactive protein, median (IQR)	234 (137–300)	272 (214–300)
Defining symptom (%)		
Dyspnea	29 (43)	
Deconditioning	27 (40)	
Cough	16 (24)	
Fatigue	9 (13)	
Joint pain	7 (10)	
Peripheral neuropathy	4 (6)	
Headache	4 (6)	
Decreased appetite	3 (4)	
Loss of taste/smell	1 (1)	
Adjustment disorder	1 (1)	
Orthostatic hypotension	1 (1)	
Cognitive impairment	1 (1)	
Median time from diagnosis to post COVID-19 condition diagnosis, days (IQR)	103 (94–125)	

*Hispanic/Latinx may be of any race.

We attempted to contact all 195 survivors telephonically. Sixty-nine patients were contacted, provided verbal informed consent, and completed the telephone survey and structured interview. Among telephone survey participants, most (89%) described not being back to their baseline health status due to physical and mental limitations. Patients described dyspnea at rest (26%) and with minimal exertion (38%). The majority had seen their primary medical doctor after C-ARDS related hospital discharge (88%) but fewer reported having been seen by a pulmonologist (42%) or having pulmonary function testing (52%). Some described lack of follow-up care over the subsequent year (11%), and subsequent emergency room visits (38%) (Table S2). Half of the patients we surveyed had an annual household income of less than $25,000 in 2019, 80% did not work at the time of the survey, and 35% reported that they struggled to pay for household expenses. Twenty-one percent reported a degree of food insecurity, and 15% noted that they skipped some medications due to cost.

Our survey revealed that 38% of respondents reported being treated disrespectfully during their hospitalization, including being treated with less courtesy and/or respect than others, receiving worse services than others, and not being listened to by a doctor or nurse. Most of this behavior was attributed by patients to race, ethnicity, or national origin (67%). Additionally, some respondents reported not receiving information in a language they understood (4%) or not having access to an interpreter when one was needed during their hospitalization (9%) (Table S2).

Patients were asked open-ended follow-up questions by interviewers to contextualize understanding of post-discharge care access and healthcare discrimination among ARDS survivors. Thematic analysis of interviews identified four perceived challenges to accessing care: decline in functional status, interpersonal discrimination during hospitalization, healthcare system inequities, and non-healthcare-related structural barriers. Sources of resilience included survivorship, faith, and family support (Table 3).

**Table 3:** Barriers to engaging in follow-up care.

Post-discharge changes in functional status	
Physical health	“I can't move. They told me I would get physical therapy and they never came. Ever since I was in the hospital, I have been unable to get out of bed. If I try, my legs shake.**” ***(non-Hispanic Black woman)* “I can’t walk. It’s hard to get to the doctor and get my medication.” (Hispanic man)*
Mental health	“[I don't seek healthcare because] I am scared they will detect something. I became traumatized after my hospitalization, and it's been difficult for me to forget.” (Hispanic man)* “[I have become] scared. I had nightmares about needles and being constantly poked.” (Hispanic man)*
Discrimination or poor treatment during care	
Interpersonal experiences	“I needed to pee, and the aide took off her gloves. I was left sitting in pee until the following day.” (Hispanic man)* “Two nurses did not want to clean me” (Hispanic woman)* “I fell out of bed in the post-discharge nursing home and nurses refused to help me.” *(non-Hispanic White woman)*
Healthcare system barriers	“I could have used more rehab, but insurance only pays for one month.” *(Hispanic woman)* “Doctors can’t always speak Spanish and interpreters are often not there when you want to communicate.” (Hispanic woman)* “Doctors' treatment is better towards White patients. I felt discriminated against when I went to the pharmacy. The pharmacist refused because she assumed I was a drug user.” *(Hispanic man)* “I felt like a Guinea pig. I felt like I was being used for monetary purposes.” *(non-Hispanic Black woman)*
Structural barriers	“My biggest concern is having to pay a $45 co-pay for each PT session.” *(non-Hispanic Black woman)* “It took a while for [the hospital] to answer my calls and get back to me, so I had to pay out of pocket to see a specialist.” *(Hispanic man)* “Money is no longer the same as it used to be. We owe 4–5 month’s rent.” (Hispanic man)* “We can’t work and are having economic difficulties but are still making ends meet. They stopped giving me discount coupons and I tried to reapply, but they denied my request because I don’t have someone to fill it out for me.” (Hispanic man)*
Sources of resilience	
“I thought my only way out was to stop existing. That's when the doctor squeezed my hand, and another thought crossed my mind: ‘I gave my family everything they needed but I never told them I loved them’. I heard the words ‘You are a survivor.’ I was ecstatic.” (Hispanic man)* “Since I got through that I feel I am a leader. Who would have thought I would survive given how sick I was?” (Hispanic man)* “I’m here because of a miracle from God. God used doctors to take care of me and if doctors are telling us to get the vaccine that means the vaccine is good.” (Hispanic male)*	

*Native language Spanish-speaker.

Regarding how physical and mental declines impacted care access, ARDS survivors described mobility issues that interfered with their ability to get to medical appointments, as well as unmet physical therapy needs. They also described ongoing impacts of their traumatic COVID-19 hospitalization, such as fear and nightmares, that caused them to avoid the healthcare system.

When asked to comment on interpersonal discrimination, such as lack of respect and inferior treatment in the hospital, C-ARDS survivors described negative interpersonal interactions with healthcare providers and neglect of physical hygiene needs. Other systemic barriers discussed included lack of access to insurance to cover outpatient services, especially rehabilitation and physical therapy. Hispanic patients described communication and language barriers, and several patients felt that providers favored white patients, and assumed that they were drug seekers. Finally, financial struggles loomed large. Patients were constrained by loss of income, out-of-pocket costs, and insufficient/non-existent insurance coverage.

Despite interpersonal and systemic level experiences of injustice, many patients also expressed sources of resilience, often in the form of faith and support networks. Individuals expressed that their faith allowed them to maintain a sense of hope and that family support was important through all stages of recovery. Finally, patients also expressed a common sense of survivorship and gratefulness for having come out the other side of this harrowing experience.

## Discussion

In our study race/ethnicity was not associated with survival to hospital discharge but was associated with health insurance status and access to rehabilitation. Like prior literature, uninsured patients were more likely to be Hispanic and discharged home rather than to a rehabilitation facility. Perceived discrimination within the healthcare setting was reported by a subset of respondents. Structural and healthcare-related barriers rooted in racism, ethnicity, and poverty were perceived as factors impacting access to post-COVID-19 care.

In comparison to other studies where racial and ethnic minorities were less likely to meet criteria for Long COVID^35^ our study of post COVID-19 ARDS survivors identified a population whose symptoms are synonymous with Long COVID, most defined by respiratory symptoms including dyspnea and cough. Long COVID symptoms resulted in functional declines that impaired ARDS survivors’ ability to receive follow-up care. However, it is uncertain how many have received this diagnosis. Since the identification of Long COVID depends on follow-up care, lack of outpatient healthcare access means that this diagnosis may not occur in this patient population, and that they also may be underrepresented in estimates of the prevalence of Long COVID. Early data on Long COVID demographics is largely white and female, suggesting that this may be a by-product of healthcare access^36^. While much remains unknown or poorly understood about Long COVID, this diagnosis is recognized under the ADA, thus opening the possibility of benefits for these patients. However, without access to healthcare and this diagnosis, this patient population is at risk for systematic exclusion.

The mental health impacts of COVID-19 have been widely reported^37^. Among C-ARDS survivors in this study, these were compounded by the trauma of their hospitalization. These experiences also contributed to an aversion to the healthcare setting, often based on fear of rehospitalization. Humiliating, hostile, and uncaring experiences were often attributed to race, ancestry, or national origins, in a context where patients felt that doctors’ treatment of white patients was better. ARDS survivors described feelings of abandonment, neglect, and distrust that they would be well-cared for and attended to.

Strengths of this study include in-depth analysis of initial COVID-19 hospitalizations for a critically ill C-ARDS cohort, complemented by comprehensive longitudinal follow-up of a racially and ethnically diverse population of C-ARDS survivors that encompasses both quantitative data and qualitative interviews. However, our findings are limited by its focus on a single hospital system, the use of non-standardized questions, and a relatively small sample size.

## Conclusion

We advocate for equity in access to follow-up medical care among COVID-19 ICU survivors through targeted policies and resource mobilization toward the communities that have been disproportionately impacted by COVID-19. Partnering between large healthcare networks and community-based organizations for the establishment of clinics and programs specifically designed for caring for discharged ICU patients, noting that these patients are at higher-than-average risk for readmission. Changes in policy expanding access to post-hospitalization rehabilitation and medical services. If non-white ARDS survivors are systematically excluded from benefits attached to a Long COVID diagnosis, this is an unacceptable social justice issue impacting a group that is already facing multiple challenges to accessing care.

### Supplementary Information

Supplementary Tables.

## Data Availability

The datasets generated during the current study are not publicly available to maintain participant confidentiality but are available from the corresponding author on reasonable request.
