# Supplementary material for: Racial and ethnic disparities post-hospitalization for COVID-19: barriers to access to care for survivors of COVID-19 acute respiratory distress syndrome
Source: Sci Rep. 2024 May 21;14:11556. doi: 10.1038/s41598-024-61097-0 (PMC11109289; doi:10.1038/s41598-024-61097-0)
Supplement: Supplementary file 1 — Supplementary Tables. [file 41598_2024_61097_MOESM1_ESM.docx]

**Table S1.** Logistic regression model identifying risk factors for 90-day mortality.

| Variable |  | **Univariate**  **OR (95% CI)** | ***p-value*** | **Multivariable**  **aOR (95% CI)** | ***p-value*** |
| --- | --- | --- | --- | --- | --- |
| Age |  | 1.06 (1.05-1.08) | <0.0001 | 1.06 (1.04-1.08) | <0.0001 |
| Gender | Female  Male | Ref  1.22 (0.84-1.79) | 0.3 | Ref  1.74 (1.10-2.77) | 0.019 |
| Obese | No  Yes |  | 0.51 | Ref  1.50 (0.86-2.44) | 0.17 |
| Hypertension | No  Yes | Ref  2.62 (1.76-3.90) | <0.001 | Ref  1.16 (0.73-1.82) | 0.17 |
| Diabetes  Mellitus | No  Yes | Ref  1.65 (1.15-2.39) | 0.007 | Ref  1.16 (0.73-1.82) | 0.53 |
| C-reactive protein |  | 0.998 (0.996-1.00) | 0.039 | 0.997 (0.994-0.999) | 0.007 |
| Race | White*  Black*  Hispanic/Latino | Ref  0.54 (0.26-1.09)  0.58 (0.32-1.08) | 0.083  0.085 | Ref  0.80 (0.35-1.80)  0.83 (0.42-1.67) | 0.58  0.61 |

*Non-Hispanic

**Table S2**. Post-Covid-9 survey responses.

| ***Post COVID-19 function*** | |
| --- | --- |
| Since your hospitalization due to COVID-19, are you back at your functional baseline? | 8 (11%) |
| What is the nature of your limitations?  Physical  Mental  Physical & Mental | 26 (38%)  2 (3%)  33 (48%) |
| Have you felt short of breath since leaving the hospital? | 46 (68%) |
| Since your hospitalization due to COVID-19, have you been hospitalized again? | 14 (20%) |
| ***Post COVID-19 Healthcare Access*** | |
| Since your hospitalization due to COVID-19, have you:  seen a primary care doctor?  visited the emergency room?  seen a pulmonary doctor? | 61 (88%)  27 (38%)  29 (42%) |
| Do you put off or neglect going to the doctor or the clinic? | 16 (23%) |
| **Financial Barriers and Economic Struggles** | |
| How hard is it for you to get the medications you are prescribed?  Not at all  Somewhat or very | 50 (73%)  10 (15%) |
| In the last month, did you skip medications to save money?  Sometimes  Never | 7 (10%)  53 (77%) |
| In the last 7 days, did you do any work for pay? | 22 (32%) |
| In the last 7 days, how difficult has it been for your household to pay for usual household expenses?  Not at all/ a little  Somewhat/very | 45 (68%)  25 (35%) |
| In the last 7 days, which of these statements best describes the food eaten in your household?  Enough of the kinds of food we wanted to eat  Enough, but not always what we wanted  Sometimes/often not enough | 43 (61%)  13 (18%)  15 (21%) |
| In 2019 what was your total household income before taxes?  <25,000  25,000-34,999  35,000-74,999  75,000 and above  Prefer not to answer | 30 (51%)  11 (19%)  11 (19%)  7 (12%)  12 (17%) |
| **Discrimination in the healthcare setting** | |
| When you seek healthcare, do you get the information in the language that you know best?  Always  Sometimes/never | 65 (94%)  4 (5%) |
| When you were hospitalized, did you have access to an interpreter if you needed one?  Always  Sometimes  Not Needed | 35 (51%)  6 (9%)  28 (41%) |
| When getting health care, how often has each experience happened to you? (mean (SD)  Treated with less courtesy than others?  Treated with less respect than others?  Received poorer services than others?  Had a doctor or nurse act as if he or she thinks you were not smart?  Had a doctor or nurse act as if he or she was afraid of you?  Had a doctor or nurse act as if he or she was better than you?  Felt like a doctor or nurse was not listening to what you were saying?  Any perceived discrimination | 16 (23%)  14 (20%)  16 (23%)  11 (16%)  2 (3%)  9 (13%)  14 (20%)  26 (38%) |
| What do you think is the main reason for the experiences you just told me about?  race, ancestry, or national origins  gender  physical disability  language  Staff neglect | 46 (67%)  4 (6%)  7 (10%)  2 (3%)  2 (3%) |
